# The Australian 'FORM' approach to guideline development: The quest for the perfect system

**DOI:** 10.1186/1471-2288-11-17

**Published:** 2011-02-15

**Authors:** Philipp Dahm, Benjamin Djulbegovic

**Affiliations:** 1 Department of Urology and Prostate Disease Center, University of Florida, College of Medicine, Gainesville, FL, USA; 2 Division and Center for Evidence-Based Medicine and Health Outcomes Research, University of South Florida & H. Lee Moffitt Cancer Center & Research Institute, Tampa, FL, USA

## Abstract

**Background:**

Clinical practice guidelines have been defined as systematically developed statements to assist practitioner and patient decision-making about appropriate health care for specific clinical circumstances. They play an important role in guiding evidence based clinical practice. The Australian National Health and Medical Research Council has developed and pilot-tested a new framework for guideline development, the FORM approach, the role of which has yet to be further defined.

**Methods:**

We critically review the elements of the FORM approach and compare it to other, more established methods for rating the quality of evidence and strength of recommendations.

**Results:**

FORM recognizes five factors that impact the strength of a recommendation which are the evidence base, consistency, clinical impact, generalizability and applicability. Consideration of these elements leads to a four-tiered rating system represented by the letters A ("body of evidence can be trusted to guide practice") to D ("body of evidence is weak and recommendation must be applied with caution"). It builds on other existing guideline methodologies such as those developed by the Scottish Intercollegiate Guidelines Network (SIGN), the Strength of Recommendation Taxonomy (SORT) and the Grading of Recommendations Assessment, Development and Evaluation (GRADE) groups. FORM distinguishes itself from other systems by its strong emphasis on applicability, which is separated out as its own category and relates the relevance of the body of evidence to the Australian healthcare system.

**Conclusions:**

The FORM approach offers a methodologically rigorous alternative approach to guideline development that places particular emphasis on aspects of applicability. This feature is unique and may prompt future adoption by other guidelines systems

## Commentary

Clinical practice guidelines have been defined as systematically developed statements to assist practitioner and patient decision-making about appropriate health care for specific clinical circumstances[1]. Alongside with efforts to systematically draw together the entire body of evidence for a specific clinical question as promoted by the Cochrane Collaboration[2] and the evidence-based medicine movement with its emphasis on critical appraisal[3], the guideline movement has been one of the driving forces towards a more evidence-based practice of medicine. Clinical practice guidelines also hold a prominent position in the hierarchy of evidence-based resources, as they link evidence with decision-making for a given clinical condition at the point of care[4].

Since their humble beginnings in the early nineties, the defining characteristics of clinical practice guidelines that can rightfully consider themselves "evidence-based" have increasingly been developed[5]. These include a formal rating of the quality of the evidence that goes beyond study design alone and considers to what extent methodological safeguards against bias (such as allocation concealment, blinding, drop-out rates etc) are put in place to minimize the risk of bias. Early on, there was little consensus on how to rate the quality of evidence, and by 2002 there were 106 competing evidentiary systems available[6]. However, basing evidentiary rules on study design alone yielded unsatisfactory results when it came to guiding the action for clinical decision-makers, thereby promoting the development of a new generation of systems to develop clinical practice guidelines[7]. This generation of methodological frameworks is represented by those currently used by the U. S. Preventive Services Task Force (USPSTF), the National Institute for Health and Clinical Excellence (NICE), the Scottish Intercollegiate Guidelines Network (SIGN), the Strength of Recommendation Taxonomy (SORT) and the Grading of Recommendations Assessment, Development and Evaluation (GRADE) groups. A major contribution of these systems has been the recognition that factors other than the quality of evidence alone impact clinical recommendations, thereby prompting a clear separation of the quality of evidence from the strength of a recommendation.

The FORM framework represents a new arrival of an evidence-based methodology to develop clinical practice guidelines[8]. It clearly acknowledges its roots in the SIGN and SORT systems, which were adapted to meet the perceived needs of stakeholder organization representatives in the Australian healthcare system. In brief, it recognizes five factors that impact the strength of a recommendation which are the evidence base, consistency, clinical impact, generalizability and applicability. Consideration of these elements then leads to a four-tiered rating system represented by the letters A ("body of evidence can be trusted to guide practice") to D ("body of evidence is weak and recommendation must be applied with caution").

Although this system is novel, it should be recognized that it differs little from the existing guidelines systems. For example, when comparing FORM with GRADE, which is used by more than 55 organizations in 23 countries, "clinical impact" refers to the likely benefit that application of the guideline can realize while also taking into account the relevance of the effect to patients (clinical importance), precision and effect size[9]. GRADE considers all of these elements in operationally different ways- it starts with the clinical importance of the outcomes, takes into account the magnitude of the effect and its precision as part of the evaluation of quality of evidence and assesses the ratio of benefit to harm (which GRADE considers one of three other dimensions distinct from the quality of evidence) in formulation of the guideline recommendations[10]. However, what distinguishes FORM from other systems is its strong emphasis on applicability, which is separated out as its own category and relates the relevance of the body of evidence to the Australian healthcare system. This feature is unique and may prompt future adoption by other guidelines systems.

In an ideal world, guidelines developers would employ a unified system to rate the quality of evidence and strengths of recommendations[11]. Doing so would dispel the "Babylonian confusion" among users trying to make sense of the varying terminology and definitions used by various guidelines developers, ultimately helping to enhance guidelines implementation[12]. To date, no such unified system exists and we are confronted with a fairly large number of competing systems that fail to readily translate one into another[13].

How should we arrive at the "best" system? To do so, one would ultimately like to show that a) the system results in making recommendations that will lead to better outcomes than recommendations by other systems and b) the system is more reproducible than others. The first point will be difficult to prove empirically and may therefore remain forever unresolved. The second point relates to the issue of whether a system such as FORM can be operationalized in terms of practical, reproducible policy and procedures. To illustrate the challenge, consider the number of combinations that can arise from the FORM system that (as shown in table 1 in the manuscript) distinguishes between five factors that can be rated in four different ways thereby resulting in 4^5 ^= 1024 combinations. These must be considered in conjunction with recommendations that are made using a four-tiered scale for or against an intervention (4^2 ^= 16 combinations). This results in a mind-boggling 16,384 (1024 × 16) ways in which a body of evidence can theoretically be categorized to support clinical recommendations. It is, however, highly likely that some combinations are more prevalent than others making development of the guidelines system more feasible than these theoretical calculations appear to indicate. Nevertheless, it is also likely that this complexity, hitherto only implicitly acknowledged by the people in the field, drives the efforts to develop new systems for guidelines development. It is also clear that as we strive to develop a unified guidelines system, we must find a way to rate a body of evidence and strength of recommendations in a reproducible and reliable manner. We believe that the most important next step in the EBM field relates to the need to perform empirical methodological research to evaluate which of the existing guidelines systems is most reproducible and performs best in the hands of the individuals they are meant to serve. Without undertaking this research, the entire evidence-based medicine edifice may lose its solid ground, built so carefully over the last 20 years.

## Pre-publication history

The pre-publication history for this paper can be accessed here:

http://www.biomedcentral.com/1471-2288/11/17/prepub
